# Physicians’ responses to computerized drug interaction alerts with password overrides

**DOI:** 10.1186/s12911-015-0194-y

**Published:** 2015-08-28

**Authors:** Yasuyuki Nasuhara, Ken Sakushima, Akira Endoh, Reona Umeki, Hiromitsu Oki, Takehiro Yamada, Ken Iseki, Makoto Ishikawa

**Affiliations:** Division of Hospital Safety Management, Hokkaido University Hospital, Sapporo, Japan; Department of Regulatory Science, Hokkaido University Graduate School of Medicine, Sapporo, Japan; Division of Medical Information Planning, Hokkaido University Hospital, Sapporo, Japan; Department of Pharmacy, Hokkaido University Hospital, Sapporo, Japan; Laboratory of Clinical Pharmaceutics and Therapeutics, Faculty of Pharmaceutical Sciences, Hokkaido University, Sapporo, Japan

**Keywords:** Adverse drug event, Alert fatigue, Computerized drug-drug interaction alert system, Contraindications for coadministration

## Abstract

**Background:**

Although evidence has suggested that computerized drug-drug interaction alert systems may reduce the occurrence of drug-drug interactions, the numerous reminders and alerts generated by such systems could represent an excessive burden for clinicians, resulting in a high override rate of not only unimportant, but also important alerts.

**Methods:**

We analyzed physicians’ responses to alerts of relative contraindications and contraindications for coadministration in a computerized drug-drug interaction alert system at Hokkaido University Hospital. In this system, the physician must enter a password to override an alert and continue an order. All of the drug-drug interaction alerts generated between December 2011 and November 2012 at Hokkaido University Hospital were included in this study.

**Results:**

The system generated a total of 170 alerts of relative contraindications and contraindication for coadministration; 59 (34.7 %) of the corresponding orders were cancelled after the alert was accepted, and 111 (65.3 %) were overridden. The most frequent contraindication alert was for the combination of 3-hydroxy-3-methylglutaryl–coenzyme A reductase inhibitors and fibrates. No incidents involving drug-drug interactions were reported among patients who were prescribed contraindicated drug pairs after an override.

**Conclusions:**

Although computerized drug-drug interaction alert systems that require password overrides appear useful for promoting medication safety, having to enter passwords to override alerts may represent an excessive burden for the prescribing physician. Therefore, both patient safety and physicians’ workloads should be taken into consideration in future designs of computerized drug-drug interaction alert systems.

## Background

Numerous widespread patient safety issues were identified in a report published by the Institute of Medicine in 2000 entitled “To Err is Human” [1]. Many other reports have shown that among all medical incidents that occur in hospitals, drug-related incidents are the most prevalent [2–4]. Gandhi et al. reported that 25 % of the outpatients at four adult primary care practices experienced adverse drug events (ADEs) in ambulatory care; 13 % of these events were serious, 28 % were ameliorable, and 11 % were preventable [5]. Unlike ADEs, which are often unpredictable, drug-drug interactions (DDIs) can be avoided if physicians take extra precautions when prescribing medications. Although evidence has shown that a computerized DDI alert system (DIAS) could reduce the incidence of DDIs [6–10], the burden of numerous reminders and alerts may cause clinicians to override not only unimportant, but also important alerts [8, 11]. Some recent studies concluded that over-alerting and a lack of practical management or recommendations often cause physicians to disregard even serious alerts [12–14]. One review article concluded that between 49 % and 96 % of computerized DDI alerts are routinely ignored or overridden [15]. This “alert fatigue” strongly limits the applicability of automated alerts. A systematic review showed that conditions such as low specificity, low sensitivity, and unclear information content may make the alert system prone to errors that result in active failures of the physician, such as ignoring important alerts, misinterpretation, and incorrect handling [15]. With the goal of reducing alert fatigue, a number of studies have attempted to identify and rate key DDIs [16, 17]. Findings from these studies suggest that physicians typically override alerts for the following three reasons: prior awareness of the DDI; insufficient knowledge of the DDI; or carelessness.

The DIAS at Hokkaido University Hospital has a very unique function in that when an alert of contraindications for coadministration is triggered, the physician must contact the hospital pharmacist to obtain a password; these passwords are randomly generated and changed daily. Next, to complete the order, the physician must enter the password along with a reason for the override. Therefore, physicians can override alerts only after considering the associated DDIs. To our knowledge, this is the first study to investigate override rates among physicians with awareness of DDIs using a DIAS that requires a password override.

To clarify physicians’ responses, we examined all DDI alerts generated for one year at Hokkaido University Hospital. In addition, to utilize more homogenized subjects, we examined only alerts generated due to relative contraindications and contraindications for coadministration as described in drug package inserts. We also examined the profiles of DDIs with respect to drug types and the presence or absence of incidents involving DDIs.

## Methods

### Drug-drug interaction database at Hokkaido University Hospital

The DDI database at Hokkaido University Hospital was developed by the institution’s pharmacy department. In Japan, drug package inserts must include the following information in relation to DDIs: 1) possible DDIs with other drugs have been considered; 2) the drug should not be coadministered with other drugs because serious DDIs may occur (relative contraindications for coadministration); and 3) coadministration of the drug with other drugs is prohibited due to the high prevalence of serious DDIs (contraindications for coadministration). The latter two points were manually entered into the computerized DIAS by the pharmacy department. Drug pairs in which contraindications were listed in the package insert of one drug but not the other were also included. The database was updated as new drugs were approved and drug package inserts were revised.

### Order entry system at Hokkaido University Hospital

**Fig. 1:**
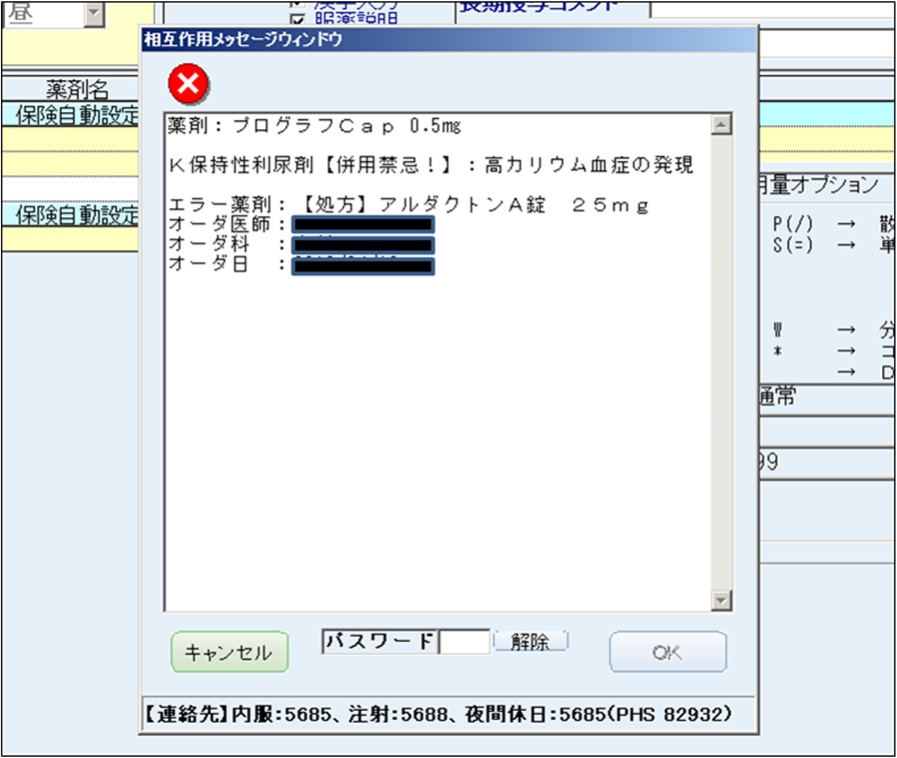
The pop-up window displayed on the screen in real time when an alert of relative contraindications and contraindications for coadministration is generated. A “Contraindication!” message is displayed on the screen along with the reason for the alert. The protocol for requesting a password is also shown

**Fig. 2:**
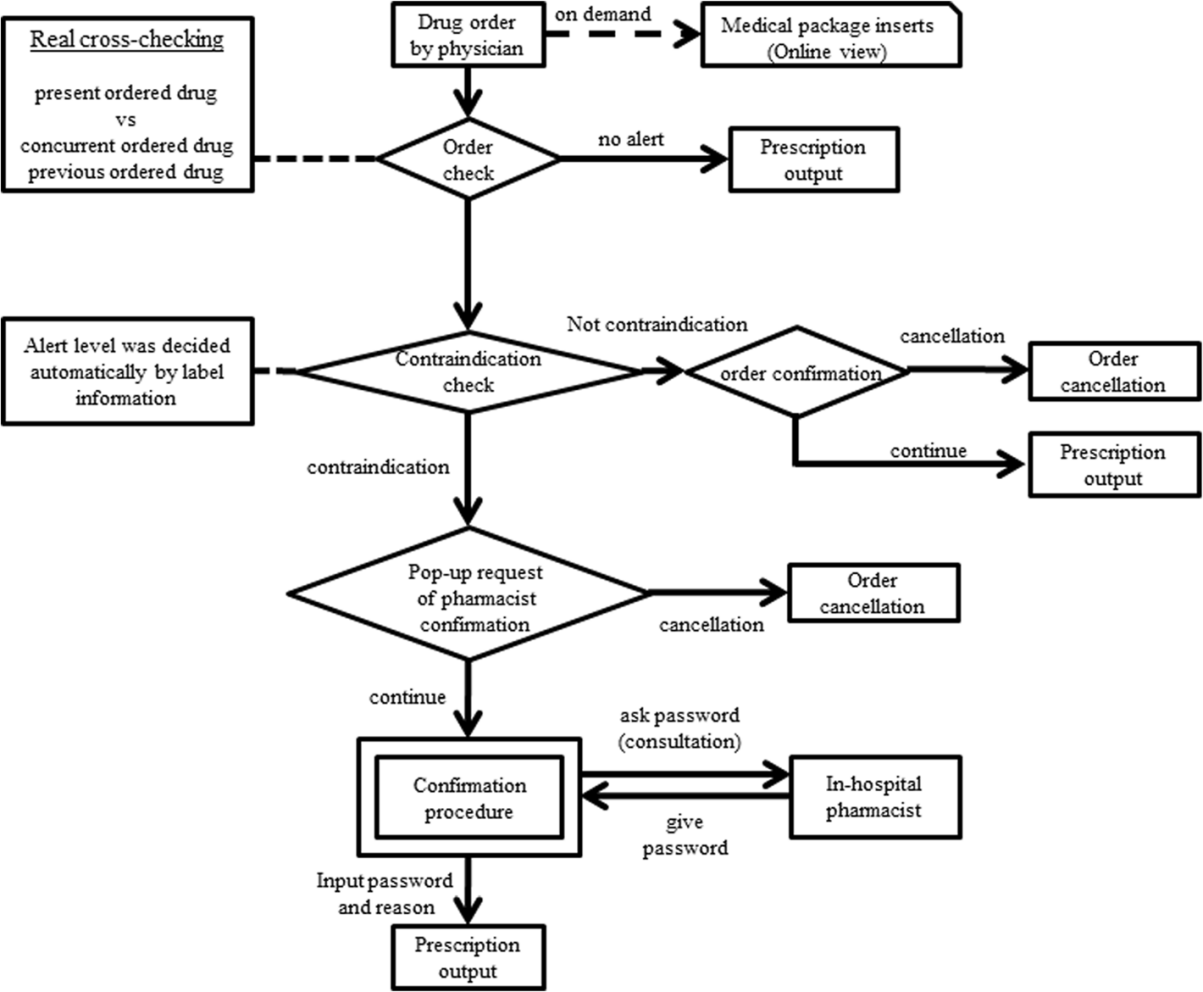
A flow chart showing the computerized drug-drug interaction alert system used at Hokkaido University Hospital

The DIAS at our hospital was originally developed by the Division of Medical Information Planning at Hokkaido University Hospital in collaboration with NEC (Nippon Denki). A computerized DIAS programmed with .NET and Visual Basic was incorporated into the existing order entry system. When a DDI is detected through cross-checking prescriptions with the DDI database, a pop-up alert window, as shown in Fig. 1 (in Japanese), is displayed in real time. An alert is triggered upon occurrence of any of the following situations: interactions with prescriptions ordered during the same visit; interactions with prescriptions ordered by different physicians; or interactions with previous prescriptions that are still active. In the case of relative contraindications and contraindications for coadministration, a “Contraindication!” message, accompanied by the reason for the alert, is displayed on the screen. If the physician wishes to override the alert and continue the order, they must first enter a password. To obtain the password, which is randomly generated and changed daily, they have to contact the hospital pharmacists. To proceed with the order, physicians must also enter the reason for overriding the alert by clicking corresponding buttons displayed on the monitor; these reasons include a long interval to avoid DDI, an expected DDI, an emergency situation in which the order is necessary, and planned use of a modified dosage to avoid the DDI. A flow chart of the computerized DIAS used at our hospital is shown in Fig. 2.

### Data collection and analysis

Hokkaido University Hospital is a national university hospital in Japan with 936 beds, about 3,000 outpatients/day, about 550 doctors, and an average of 37,500 prescriptions/month. All drugs prescribed at our hospital are ordered through the computerized order entry system and recorded in the database; all DDI alerts triggered by the system are also recorded. All DDI alerts triggered by the computerized DIAS for prescriptions from Hokkaido University Hospital between December 2011 and November 2012 were included in this study (268,622 prescriptions for the study period). A database specialist (RU) from the Division of Medical Information Planning extracted and compiled the necessary data into a spreadsheet for analysis by two experienced (KN and YN). In April 2012 and August 2014, medical records were reviewed (YN) for the presence or absence of incidents involving DDIs in patients prescribed contraindicated drug pairs.

### Ethics

This study was approved by the Institutional Review Board of Hokkaido University Hospital.

## Results

**Fig. 3:**
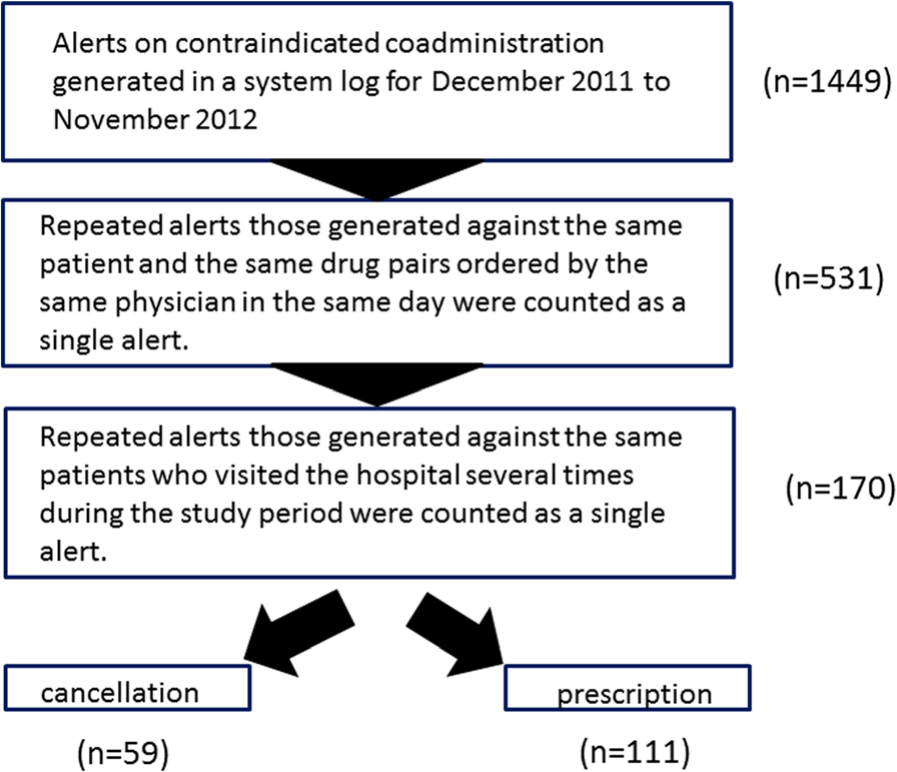
The number of alerts of the coadministration for contraindicated drug pairs generated in this study. Alerts are categorized according to the reasons described in the figure

For one year from December 2011 to November 2012, 1449 alerts for contraindicated coadministration were recorded in the system log (Fig. 3). Among these alerts, some were generated for the same patient and the same drug pairs ordered by the same physician on the same day. Physicians had two typical response patterns when making repeated attempts to reorder the prescription. The first response primarily involved physicians who were unfamiliar with the DIAS. These physicians thought that they could override DDI alerts simply by clicking the “cancel” button. Once discovering that their order had actually been cancelled, they would reorder the drugs and contact the hospital pharmacist to obtain a password. The other common response by physicians was to purposefully cancel their order when a DDI alert was triggered. Then, after careful consideration, they would then reorder the same pair of contraindicated drugs and contact the pharmacist to obtain a password. When these types of repeated alerts were counted as single alerts, the number of alerts for the study period dropped from 1449 to 531. In addition, some patients visited the hospital several times during the study period. Therefore, alerts generated for the same drug pair for the same patient by the same physician were also counted as single alerts; this further reduced the number of alerts generated during the study period to 170.

In response to these 170 alerts, 59 (34.7 %) of the corresponding orders were cancelled due to alert acceptance, and 111 (65.3 %) were prescribed after a password override (Fig. 3). Drug pairs for which physicians overrode DDI alerts more than five times are listed in Table 1. The most frequent contraindication alert was for the combination of HMG-CoA (3-hydroxy-3-methylglutaryl–coenzyme A) reductase inhibitors and fibrates, followed by tacrolimus hydrate and potassium-sparing diuretics. No incidents due to these combinations were recorded in the patients’ medical records up to 28 months after the prescriptions were issued. Drug combinations that were cancelled more than twice due to alert acceptance are listed in Table 2.

**Table 1:** Drug pairs for which physicians overrode drug-drug interaction alerts more than five times

Drug pairs		Orders
HMG-CoA reductase inhibitors	Fibrates	35
Tacrolimus hydrate	Potassium-sparing diuretics	15
Triptans	Triptans^a^	8
Immunosuppressants	Immunosuppressants	7
Albumin tannate	Ferrous fumarate	7
Potassium-sparing diuretics	Potassium-sparing diuretics	
	Potassium chloride	6

^a^Coadministration of tablet and nasal inhalation or injection included

**Table 2:** Drug combinations cancelled due to alert acceptance

Drug pairs		Orders
Albumin tannate	Ferrous fumarate	10
HMG-CoA reductase inhibitors	Fibrates	8
Miconazole	Calcium channel blockers	5
Voriconazole	Triazolam	4
Triptans	Triptans^a^	4
Selegiline hydrochloride	SSRI^b^	3
Potassium-sparing diuretics	Potassium-sparing diuretics	
	Potassium chloride	3

Combinations that were cancelled more than twice are listed

^a^Coadministration of tablet and nasal inhalation or injection included

^b^SSRI: selective serotonin reuptake inhibitor

## Discussion

In the present study, the overall alert override rate was 65.3 %, which is compatible with override rates reported in previous studies between 49 % and 96 % [15]. One study reported an overall acceptance rate of only 8.5 %; however, most of the alerts were overridden (91.5 %). Regarding the recognition of DDIs in that study, physicians were aware of 82.0 % of the DDIs, were unaware of 15.9 %, and ignored 2.1 % of the alerts [18]. The present study has three important differences from previous studies. First, in order to override alerts, physicians had to contact pharmacists to obtain passwords, which were randomly assigned and changed daily. When physicians see alerts on the screen, they may intentionally or unintentionally ignore them and click the “continue” or “ignore” button. In the computerized DIAS in our hospital, the password system prevents physicians from prescribing contraindicated drug pairs carelessly. Therefore, physicians who overrode alerts and prescribed all 111 contraindicated drug pairs were aware of the contraindications. Second, we only analyzed alerts generated due to relative contraindications and contraindications for coadministration as described in drug package inserts. Third, because physicians had to consult with pharmacists directly to obtain override passwords, physicians could only decide to continue with their orders after receiving detailed pharmaceutical reasons for the contraindications. The present study therefore revealed that even under such conditions, physicians still override DDI alerts at a high rate. Furthermore, no ADEs related to DDIs were reported among patients who were prescribed contraindicated drug pairs after an alert override.

On the other hand, 34.7 % of all contraindicated orders were cancelled due to DDI alerts. In these cases, the physician may have cancelled the order because they were unaware of the contraindication before the alert. They then could have decided that the contraindicated drugs were not absolutely necessary and prescribed alternative medications. The cancelled drug pairs were not only low-risk pairs such as albumin tannate and ferrous fumarate, but also high-risk pairs such as voriconazole and triazolam (Table 2). As a result, it is also possible that alerts help prevent incidents involving DDIs. Therefore, we conclude that a computerized DIAS that requires password overrides for coadministration of contraindicated drugs is useful for promoting medication safety.

This study did have several limitations. First, we only performed quantitative analysis. In the DIAS at our hospital, physicians choose the reason for overriding an alert by clicking corresponding buttons displayed on the monitor; these reasons include a long interval to avoid DDI, an expected DDI, an emergency situation in which the order is necessary, planned use of a modified dosage to avoid the DDI, and others. To understand the behavior of physicians, it is important to analyze the frequency of selection for each reason; however, in this study, nearly all of the reasons were “others”, which may indicate negligence on the part of the physician. Therefore, we could not perform an analysis regarding the reasons for alert overrides; such an analysis should be performed in a future study. Second, detailed information about physicians, such as their clinical experience or whether they were generalists or specialists, was not obtained in this study. The influence of background characteristics and experience on physicians’ behaviors in response to alerts should be investigated in a future study. It would also be important to analyze physicians’ behaviors after obtaining detailed explanations from pharmacists. The DIAS recorded all instances in which passwords were issued, but not consultations between physicians and pharmacists; therefore, when physicians attempted to override alerts, we could not distinguish whether they had obtained detailed pharmaceutical explanations before cancelling the order. It would also be useful to obtain physicians’ voices regarding systems that require password overrides to continue an order and how such systems affect their workflow.

In a recent report, contraindicated combinations based on drug package inserts were prescribed for just 0.2 % of over 400,000 patients in a Japanese database of health insurance claims [19]. The most frequently contraindicated drug combinations in that study involved lipid-regulating drugs, anti-migraine drugs, drugs that included metal irons, antifungals, macrolides, and immunosuppressants. Therefore, the result of our study may be generalized in point of the sort of prescribed contraindicated coadministration pairs at least in Japan. Furthermore, unlike the study involving patients in the Japanese database of health insurance claims, our study investigated the presence or absence of incidents involving DDIs.

These results suggest that physicians’ workloads should be considered in future computerized DIAS designs. One method of reducing alert fatigue may be to temporarily deactivate alerts for the same contraindicated drug pairs prescribed to the same patient by the same physician that have been overridden. The results of this study shed light on possible strategies for developing a more efficient DIAS.

## Conclusions

In this study, we explored the behavior of physicians only in regard to orders requiring password overrides for the coadministration of contraindicated drug pairs in a DIAS at Hokkaido University Hospital in Japan. Based on our results and those from previous studies, a computerized DIAS with password overrides is useful for promoting medication safety. However, the password override system may represent an excessive burden for physicians and thereby increase alert fatigue. Even though physicians who were aware of contraindicated drug pairs still overrode DDI alerts at a high rate, no incidents involving DDIs were reported among patients who were prescribed contraindicated drug pairs after an override. On the other hand, 34.7 % of the contraindicated orders were cancelled due to alert acceptance. Therefore, both patient safety and physicians’ workloads should be considered in future computerized DIAS designs.

## Abbreviations

ADE: Adverse Drug Event
DDI: Drug-Drug interaction
DIAS: Drug-Drug Interaction Alert System
