# Erratum to: Physicians’ responses to computerized drug interaction alerts with password overrides

**DOI:** 10.1186/s12911-016-0347-7

**Published:** 2016-08-10

**Authors:** Yasuyuki Nasuhara, Ken Sakushima, Akira Endoh, Reona Umeki, Hiromitsu Oki, Takehiro Yamada, Ken Iseki, Makoto Ishikawa

**Affiliations:** 1 Division of Hospital Safety Management, Hokkaido University Hospital, Sapporo, Japan; 2 Department of Regulatory Science, Hokkaido University Graduate School of Medicine, Sapporo, Japan; 3 Division of Medical Information Planning, Hokkaido University Hospital, Sapporo, Japan; 4 Department of Pharmacy, Hokkaido University Hospital, Sapporo, Japan; 5 Laboratory of Clinical Pharmaceutics and Therapeutics, Faculty of Pharmaceutical Sciences, Hokkaido University, Sapporo, Japan

## Erratum

Following publication of our paper in BMC Medical Informatics and Decision Making [1], it was brought to our attention that we had used a slightly modified version of DIAS which had been originally developed by another group [2]. We inadvertently failed to include this paper in the reference list.

On page two, line 21-23 in the left column, this should have been presented as “This study investigated override rates among physicians with awareness of DDIs using DIAS that requires a password override.” On page two, line 2-4, in the right column, this should have been presented as “The DIAS that requires a password override at our hospital was originally developed in collaboration between the Department of Hospital Pharmacy and Pharmacology, Asahikawa Medical University and Techno-Forum Co. Ltd., Japan”.

As a result, some of figures and tables were similar in both papers, especially figure 1 (in Japanese) is very similar, therefore please disregard it. We sincerely apologize for this oversight.
